# Predictors of long-term survival among first-ever ischemic and hemorrhagic stroke in a Brazilian stroke cohort

**DOI:** 10.1186/1471-2377-13-51

**Published:** 2013-05-24

**Authors:** Alessandra C Goulart, Tiotrefis G Fernandes, Itamar S Santos, Airlane P Alencar, Isabela M Bensenor, Paulo A Lotufo

**Affiliations:** 1 Hospital Universitário, University of São Paulo, São Paulo, Brazil; 2 University Federal of Amazonas, Coari, Amazonas, Brazil; 3 Institute of Mathematics and Statistics, University of São Paulo, São Paulo, Brazil; 4 School of Medicine, University of São Paulo, São Paulo, Brazil; 5 Center for Clinical and Epidemiological Research, Hospital Universitario, Av. Prof Lineu Prestes 2565, Cidade Universitária, Butantan, São Paulo CEP 0550800-900, Brazil

**Keywords:** Stroke, Registry, Long-term predictors, Survival

## Abstract

**Background:**

Few studies have examined both ischemic and hemorrhagic stroke to identify prognostic factors associated to long-term stroke survival. We investigated long-term survival and predictors that could adversely influence ischemic and hemorrhagic first-ever stroke prognosis.

**Methods:**

We prospectively ascertained 665 consecutive first-ever ischemic and hemorrhagic stroke cases from “The Study of Stroke Mortality and Morbidity” (The EMMA Study) in a community hospital in São Paulo, Brazil. We evaluated cardiovascular risk factors and sociodemographic characteristics (age, gender, race and educational level).

**Results:**

We found a lower survival rate among hemorrhagic cases compared to ischemic stroke cases at the end of 4 years of follow-up (52% vs. 44%, p = 0.04). The risk of death was two times higher among people with ischemic stroke without formal education. Also, we found consistently higher risk of death for diabetics with ischemic stroke (HR = 1.45; 95% CI = 1.07-1.97) compared to no diabetics. As expected, age equally influenced on the high risk of poor survival, regardless of stroke subtype.

**Conclusions:**

For ischemic stroke, the lack of formal education and diabetes were significant independent predictors of poor long-term survival.

## Background

Although pathologic mechanisms for ischemic and hemorrhagic stroke are clearly distinct, most previous studies have failed to compare long-term prognosis including both ischemic and hemorrhagic stroke [1-5].

It has been known that hemorrhagic stroke (HS) is associated with a very high risk of death in the acute and sub-acute phase [6,7]. In the other hand, ischemic stroke (IS) is more prevalent and presents higher life expectancy than HS [5]. Despite the importance of investigating stroke survival, particularly in developing countries as Brazil that has one of the highest rates of hemorrhagic stroke in Latin America [8], publications in this field are sparse and come from developed countries [9-11].

Thus, we sought to investigate long-term survival and predictors that could influence adversely ischemic and hemorrhagic first-ever stroke prognosis along 4 years of follow-up.

## Methods

### Population and study area

Study subjects were participants of “The Study of Stroke Mortality and Morbidity” (The EMMA Study), a stroke surveillance cohort that began on April 10, 2006. A more detailed description of the study can be found elsewhere [12]. Here, we considered all consecutive first-ever stroke individuals older than 35 years of age, with a confirmed diagnosis of ischemic stroke or intracerebral haemorrhage, who were discharged after a first hospitalization for stroke at community hospital from April 2006 to December 2010. The Hospital Universitário is located in the west area of the city of São Paulo, the largest metropolitan area of South America, and it is the only facility in this area of study, which includes 420,000 inhabitants. For survival analyses, participants were followed-up from hospital admission until death or censoring until in 2010.

### Data collection

The EMMA Study was based on the standardized World Health Organization (WHO) stepwise approach to stroke surveillance [13]. All information was collected by trained interviewers and medical researchers according to the STEPS Stroke Manual Instructions [13]. Additional information including vital status during follow-up was updated through telephone contact, medical registers, and death certificates with the collaboration of the municipal and state’s health offices. Written informed consent was obtained from all potential stroke patients admitted to the hospital who agreed to participate in this study, and each subject received a copy of the consent form. Written informed consent was obtained from all potential stroke patients admitted to the hospital who agreed to participate in this study, and each subject received a copy of the consent form. The institutional review board of the Hospital Universitário of the University of Sao Paulo approved the research protocol.

### Stroke definition

Stroke was defined according to WHO criteria as “rapidly developing symptoms and/or signs of focal (or at times global), and lasting longer than 24 hours (or leading to death), and of presumed vascular origin” [13]. Each event was classified as being the patient’s “first ever in a lifetime” clinically evident stroke, confirmed by contrast computed tomography (CT) scan and neurological evaluation during hospitalization. Stroke diagnosis was validated by three of the researchers and then categorized according to stroke subtypes based on the Tenth International Classification of Diseases (ICD-10) (chapter I) as ischemic stroke (ICD-10: I63) or intracerebral haemorrhage (ICD-10: I61).

### Statistical analysis

The comparison of life table survival across 4 years of follow-up was performed using the Wilcoxon test according to main baseline sociodemographics characteristics and preclinical conditions as following: age (35-59/60-79/≥80y-old), gender, race as self-reported skin color (White, Brown and Black), educational level (illiterate, 1–7 years, ≥8 years), marital status (married, single, divorced and widowed), hypertension (yes/no), diabetes (yes/no) and stroke type (hemorrhagic and ischemic). Kaplan-Meyer curves to demonstrate survival according to main sociodemographic and cardiovascular risk factors were also assembled. We performed Cox proportional hazards survival analysis to investigate sociodemographic and cardiovascular risk factors associations, adjusting for potential confounding factors according to all stroke and stroke subtypes (IS and HS). We included in the models significantly associated variables at level <0.20. For all analyses, *P*- values less than 0.05 were also considered as significant. All the statistical analyses were performed with the statistical software SPSS version 19.0.

## Results

**Figure 1:**
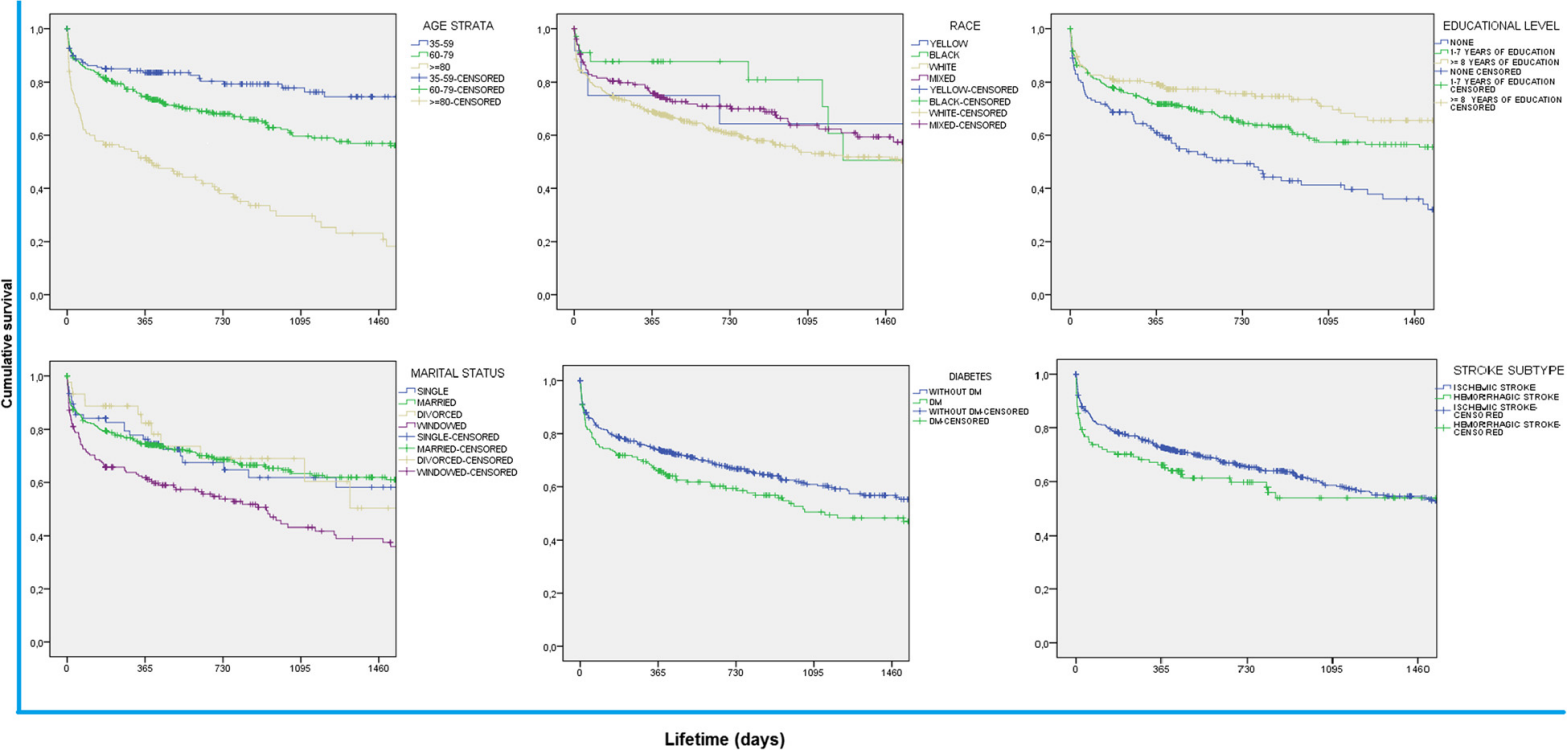
Kaplan-Meier survival curves during 4-year-follow-up according to age strata (A), race-self reported skin color (B), educational level (C), marital status (D), diabetes (E) and stroke subtype (F).

From 665 cases diagnosed as first-ever stroke enrolled in the EMMA cohort, 545 (82.6%) were identified as IS and 116 (17.4%) as HS during 4-year follow-up. Mean age was 68 years (±13.3), 53.8% of participants were male, and almost 70% had less than eight years of formal education. Overall survival rate was 48% (mean survival of 40 months) (Data not shown). Individuals aged 80 y-old or more, without formal education, widowed or diabetic at the occasion of acute event presented lower survival rates across 4-year observation period compared to other subgroups (Table 1). Kaplan-Meier curves confirmed these results (Figure 1). Multivariate regression models are shown in Table 2. Lack of formal education and diabetes were significant prognostic factors associated to higher mortality in IS subjects during follow-up. As expected, aging was also a risk factor for poorer survival, regardless of stroke subtype.

**Table 1:** Cumulative survival rates according to baseline characteristics among 665 participants from the EMMA cohort during 4-year follow-up

	**Cumulative proportion surviving (95% IC)**				
**Baseline characteristics**	**1-year**	**2- year**	**3-year**	**4-year**	***P*-values**
**Age strata**					<0.001
35-59	0.80 (0.74-0.86)	0.78 (0.70-0.86)	0.74 (0.66-0.82)	0.70 (0.60-0.79)	
60-79	0.68 (0.62-0.73)	0.60 (0.54-0.66)	0.57 (0.51-0.63)	0.55 (0.49-0.61)	
≥ 80	0.38 (0.30-0.46)	0.30 (0.20-0.39)	0.24 (0.14-0.34)	0.15 (0.05-0.25)	
**Gender**					0.48
Male	0.66 (0.60-0.72)	0.60 (0.54-0.66)	0.57 (0.51-0.63)	0.54 (0.46-0.62)	
Female	0.63 (0.57-0.69)	0.56 (0.50-0.62)	0.51 (0.45-0.57)	0.47 (0.39-0.55)	
Race/ethnicity*					
White	0.60 (0.54-0.66)	0.54 (0.48-0.59)	0.52 (0.46-0.58)	0.47 (0.41-0.53)	0.03
Brown	0.71 (0.63-0.79)	0.64 (0.56-0.72)	0.59 (0.49-0.69)	0.57 (0.47-0.67)	
Black	0.86 (0.74-0.98)	0.79 (0.61-0.97)	0.49 (0.19-0.78)	0.49 (0.19-0.78)	
**Educational level**					
Illiterate	0.49 (0.39-0.59)	0.41 (0.31-0.51)	0.36 (0.26-0.46)	0.26 (0.14-0.38)	<0.001
1-7 years	0.65 (0.59-0.71)	0.58 (0.52-0.64)	0.57(0.51-0.63)	0.56 (0.48-0.64)	
≥ 8 years	0.75 (0.69-0.81)	0.70 (0.62-0.78)	0.65 (0.57-0.73)	0.62 (0.52-0.72)	
**Marital status**					
Married	0.69 (0.63-0.75)	0.63 (0.57-0.69)	0.62 (0.56-0.68)	0.58 (0.50-0.66)	<0.001
Single	0.68 (0.56-0.79)	0.62 (0.48-0.76)	0.59 (0.45-0.72)	0.59 (0.45-0.72)	
Divorced	0.70 (0.54-0.86)	0.70 (0.54-0.86)	0.49 (0.22-0.76)	0.49 (0.22-0.76)	
Widowed	0.53 (0.45-0.61)	0.43(0.35-0.51)	0.39 (0.29-0.49)	0.34 (0.24-0.44)	
**High blood pressure**					
Yes	0.64 (0.60-0.68)	0.56 (0.50-0.62)	0.52 (0.46-0.58)	0.48 (0.42-0.54)	0.55
No	0.66 (0.63-0.71)	0.66 (0.55-0.67)	0.61 (0.51-0.63)	0.61 (0.60-0.72)	
**Diabetes**					
Yes	0.59 (0.51-0.67)	0.50 (0.42-0.58)	0.48 (0.40-0.56)	0.46 (0.38-0.54)	0.04
No	0.67 (0.63-0.71)	0.61 (0.55-0.67)	0.57 (0.51-0.63)	0.52 (0.60-0.72)	
**Stroke subtype**					
Ischemic stroke	0.66 (0.62-0.70)	0.59 (0.55-0.63)	0.55 (0.49-0.61)	0.52 (0.46-0.58)	0.04
Intracerebral hemorrhage	0.59 (0.49-0.69)	0.53 (0.43-0.63)	0.53(0.43-0.63)	0.44 (0.28-0.60)	

Overall *P*-values were obtained from Wilcoxon analysis.

*Race as defined as self-reported skin color.

**Table 2:** Predictors of poor long term survival among 665 participants from the EMMA cohort during 4 years of follow-up

**All stroke**	**1-year**	**2- year**	**3-year**	**4-year**
	**Hazard ratio (95% IC)**	**Hazard ratio (95% IC)**	**Hazard ratio (95% IC)**	**Hazard ratio (95% IC)**
**Age strata**				
35-59	Reference (1.0)	Reference (1.0)	Reference (1.0)	Reference (1.0)
60-79	1.38 (0.85-2.11)	1.52 (1.00-2.34)	1.57 (1.04-2.38)	1.46 (0.99-2.17)
≥ 80	2.92 (1.76-4.86)	3.67 (2.27-5.93)	3.66 (2.29-5.83)	3.42 (2.19-5.34)
**Race**				
White	Reference (1.0)	Reference (1.0)	Reference (1.0)	Reference (1.0)
Brown	0.79 (0.55-1.14)	0.78 (0.57-1.12)	0.81 (0.59-1.15)	0.83 (0.61-1.12)
Black	0.42 (0.15-1.14)	0.37 (0.14-1.00)	0.44 (0.18-1.08)	0.54 (0.26-1.10)
**Educational level**				
≥ 8 years	Reference (1.0)	Reference (1.0)	Reference (1.0)	Reference (1.0)
1-7 years	1.26 (0.85-1.87)	1.25 (0.87-1.81)	1.27 (0.89-1.81)	1.22 (0.87-1.71)
Illiterate	1.59 (1.03-2.48)	1.96 (1.31-2.94)	1.89 (1.28-2.81)	1.83 (1.26-2.68)
**Marital status**				
Married	Reference (1.0)	Reference (1.0)	Reference (1.0)	Reference (1.0)
Single	0.87 (0.52-1.48)	1.01 (0.63-1.62)	1.01 (0.64-1.60)	1.00 (0.63-1.54)
Divorced	0.71 (0.33-1.54)	0.82 (0.43-1.59)	0.82 (0.43-1.58)	0.98 (0.55-1.75)
Widowed	1.10 (0.77-1.57)	1.05 (0.75-1.48)	1.06 (0.76-1.46)	1.07 (0.78-1.46)
**Diabetes**				
No	Reference (1.0)	Reference (1.0)	Reference (1.0)	Reference (1.0)
Yes	1.38 (1.01-1.89)	1.45 (1.08-1.94)	1.42 (1.07-1.87)	1.41 (1.07-1.85)
**Ischemic stroke**				
**Age strata**				
35-59	Reference (1.0)	Reference (1.0)	Reference (1.0)	Reference (1.0)
60-79	1.30 (0.77-2.19)	1.38 (0.85-2.25)	1.43 (0.90-2.28)	1.35 (0.87-2.01)
≥ 80	2.90 (1.62-5.18)	3.40 (1.97-5.87)	3.37 (1.99-5.71)	3.21 (1.94-5.31)
**Race**				
White	Reference (1.0)	Reference (1.0)	Reference (1.0)	Reference (1.0)
Brown	0.85 (0.57-1.26)	0.84 (0.58-1.22)	0.86 (0.61-1.22)	0.88 (0.63-1.22)
Black	0.52 (1.90-1.42)	0.45 (1.66-1.23)	0.45 (0.17-1.24)	0.57 (2.64-1.23)
**Educational level**				
≥ 8 years	Reference (1.0)	Reference (1.0)	Reference (1.0)	Reference (1.0)
1-7 years	1.32 (0.83-2.08)	1.47 (0.95-2.27)	1.48 (0.98-2.23)	1.41 (0.95-2.10)
Illiterate	1.57 (0.94-2.63)	2.12 (1.31-3.44)	2.00 (1.27-3.21)	2.00 (1.28-3.10)
**Marital status**				
Married	Reference (1.0)	Reference (1.0)	Reference (1.0)	Reference (1.0)
Single	0.71 (0.37-1.36)	0.83 (0.47-1.49)	0.84 (0.49-1.47)	0.83 (0.48-1.42)
Divorced	0.64 (0.27-1.48)	0.81 (0.40-1.64)	0.80 (0.40-1.61)	0.98 (0.53-1.80)
Widowed	1.10 (0.74-1.63)	1.04 (0.71-1.52)	1.04 (0.73-1.49)	1.06 (0.75-1.50)
**Diabetes**				
No	Reference (1.0)	Reference (1.0)	Reference (1.0)	Reference (1.0)
Yes	1.49 (1.05-2.12)	1.54 (1.11-2.16)	1.48 (1.09-2.03)	1.45 (1.07-1.97)
**Intracerebral hemorrhage**				
**Age strata**				
35-59	Reference (1.0)	Reference (1.0)	Reference (1.0)	Reference (1.0)
60-79	1.65 (0.61-4.42)	2.16 (0.83-5.61)	2.40 (0.94-6.14)	2.27 (0.93-5.53)
≥ 80	3.44 (1.11-10.64)	3.53 (1.19-10.46)	3.83 (1.35-10.85)	3.36 (1.23-9.18)
**Race**				
White	Reference (1.0)	Reference (1.0)	Reference (1.0)	Reference (1.0)
Brown	0.55 (0.21-1.42)	0.59 (0.25-1.37)	0.56 (0.25-1.29)	0.57 (0.25-1.31)
Black	-	-	0.48 (0.06-3.78)	0.51 (0.07-3.97)
**Educational level**				
≥ 8 years	Reference (1.0)	Reference (1.0)	Reference (1.0)	Reference (1.0)
1-7 years	1.08 (0.48-2.46)	0.81 (0.37-1.77)	0.76 (0.35-1.62)	0.70 (0.33-1.49)
Illiterate	1.64 (0.65-4.14)	1.68 (0.75-3.77)	1.64 (0.75-3.57)	1.41 (0.65-3.06)
**Marital status**				
Married	Reference (1.0)	Reference (1.0)	Reference (1.0)	Reference (1.0)
Single	1.53 (0.56-5.17)	1.86 (0.74-4.66)	1.77 (0.74-4.25)	1.84 (0.78-4.37)
Divorced	2.16 (0.27-17.53)	1.13 (0.15-8.67)	1.20 (0.16-9.18)	1.21 (0.16-9.25)
Widowed	1.26 (0.50-3.13)	1.45 (0.63-3.33)	1.46 (0.67-3.21)	1.42 (0.65-3.14)
**Diabetes**				
No	Reference (1.0)	Reference (1.0)	Reference (1.0)	Reference (1.0)
Yes	0.95 (0.46-1.94)	1.02 (0.52-1.97)	1.01 (0.53-1.92)	1.09 (0.58-2.06)

*P*-values were obtained from Cox Regression. CI 95%: confidence interval 95%. All multivariate analyses were adjusted for age, race, educational level, marital status and diabetes, except itself.

## Discussion

Overall, life expectancy within the first 4 years after stroke was about 50% in The EMMA Study. Our cumulative surviving rate for hemorrhagic stroke was about 44%, representing a relatively higher probability of long-term survival compared to other hospital registry with HS patients performed in Europe [6]. Despite this fact, we confirmed our previous finding [7] of higher risk of death for hemorrhagic stroke (HS) compared to ischemic stroke (IS) even after a 4-year follow-up. It contrasts to the findings from Andersen et al.’s study [9], which found no differences on mortality according to stroke subtypes after three months in the Nationwide Danish stroke hospital registry. These dissimilarities may be explained by population or long-term care differences between countries. Regarding to ischemic stroke survival, our findings were consistent with other population-based studies in developed countries [3-5,10].

Ischemic stroke patients with no educational degree kept higher risk of death with four years after the acute event compared to those individuals with 8 or more years of schooling in our cohort. Further, after 2 years of observation, diabetes increased the risk of dying about 1.5 times among ischemic stroke patients.

Adjustment for potential confounders did not attenuate risk due to educational attainment among ischemic stroke individuals. In contrast, we observed a significant two-fold increase in the risk of fatal IS, but not HS, among illiterate individuals 4 years after an acute event. Point estimates, however, showed a trend for poorer prognosis in the HS subgroup. In the same direction, a retrospective study using mortality data also in the city of Sao Paulo, Brazil [14], found a positive association between lower socio-economic status and mortality in both stroke subtypes. Similar to our previous published findings among ischemic stroke patients [15], we found high rates of moderate to severe disability, as well as, tobacco consumption among less educated patients, which could have contributed indirectly to a lower survival in this subset of patients compared to those more educated. We can also speculate that patients with low education have a more difficult access to treatment and rehabilitation after emergency care. Although the city of São Paulo has been the largest and richest city in the country, it is very heterogeneous and Butantan has a great number of favelas with precarious living conditions and no public transportation available to individuals who require special care as post stroke patients. Beyond that, not all primary care units have teams that routinely assisted patients with disabilities at home. The possible consequence is that patients with low education and a high degree of disabilities could be restricted to bed with very limited or no access to medical treatment and rehabilitation. Probably, we may be able to confirm these findings in our cohort with longer follow-up or the inclusion of more cases of HS. Also, the effect of diabetes on the risk of death particularly among ischemic stroke patients was consistent. Similar findings, but with shorter follow-up, was described in a Nationwide Danish Study [16]. Our main limitation was the single center design with few cases of hemorrhagic stroke, which made it difficult to perform additional analysis in this subgroup. This study has also some strength. This is a long-term cohort of stroke patients in Latin America, a less studied scenario. Moreover, we included both stroke subtypes which are not often investigated in other hospital-based stroke registries. Finally, there is no difference regarding to post-stroke treatment comparing survivors and those who died. All consecutive patients who are attended in the Hospital Universitário of the University of São Paulo (HU-USP) and agreed to participate of the EMMA cohort received same acute care for stroke depending on whether it is ischemic or hemorrhagic. Although, the hospital does not have a stroke Unit, the HU-USP is a teaching community hospital with a 280-bed facility, which offers a good support for emergencies and is responsible for 80% of the hospitalizations of people living in this location. After emergency care, patients are referred to public primary care; however, most services cannot offer rehabilitation treatment for all of them. As a consequence, patients with low socioeconomic levels do not have access to rehabilitation post-stroke. In a secondary analysis, we found more than 70% of patients without rehabilitation after stroke in our cohort. Further, we found no differences in long-term rehabilitation treatment comparing survivors and those died along 4-year follow-up.

## Conclusions

For ischemic stroke, the lack of formal education and diabetes, besides aging, were significant independent predictors of poor long-term survival.

## Abbreviations

HS: Hemorrhagic stroke; IS: Ischemic stroke; The EMMA Study: The Study of Stroke Mortality and Morbidity; WHO: World Health Organization; CT: Computed tomography; ICD-10: Tenth International Classification of Diseases.

## Competing interests

Dr Lotufo and Dr Bensenor are recipient of a grant for established investigator from Conselho Nacional de Pesquisa (CNPq), Brasília, Brazil.

## Authors’ contributions

ACG has made substantial contributions to conception and design, or acquisition of data, or analysis and interpretation of data; have been involved in drafting the manuscript or revising it critically for important intellectual content and have given final approval of the version to be published. TGF have made substantial contributions to conception and design, or acquisition of data, or analysis and interpretation of data; have been involved in drafting the manuscript or revising it critically for important intellectual content and has given final approval of the version to be published. ISS has been involved in drafting the manuscript or revising it critically for important intellectual content and have given final approval of the version to be published. APA has made substantial contributions to conception and design, or acquisition of data, or analysis and interpretation of data and have given final approval of the version to be published. PAL has been involved in drafting the manuscript or revising it critically for important intellectual content and has given final approval of the version to be published. IMB has been involved in drafting the manuscript or revising it critically for important intellectual content and has given final approval of the version to be published.

## Pre-publication history

The pre-publication history for this paper can be accessed here:

http://www.biomedcentral.com/1471-2377/13/51/prepub
